# Assessment of the Cardiac Functions Using Full Conventional Echocardiography with Tissue Doppler Imaging before and after Xylazine Sedation in Male Shiba Goats

**DOI:** 10.3390/ani10122320

**Published:** 2020-12-07

**Authors:** Ahmed S. Mandour, Haney Samir, Tomohiko Yoshida, Katsuhiro Matsuura, Hend A. Abdelmageed, Mohamed Elbadawy, Salim Al-Rejaie, Hussein M. El-Husseiny, Ahmed Elfadadny, Danfu Ma, Ken Takahashi, Gen Watanabe, Ryou Tanaka

**Affiliations:** 1 Department of Animal Medicine (Internal Medicine), Faculty of Veterinary Medicine, Suez Canal University, Ismailia 41522, Egypt; 2 Laboratory of Veterinary Surgery, Tokyo University of Agriculture and Technology, Tokyo 183-8509, Japan; tomohiko7731-yoshida@yahoo.co.jp (T.Y.); k.matsuura.vet@gmail.com (K.M.); hussien.alhussieny@fvtm.bu.edu.eg (H.M.E.-H.); dandanma1000@gmail.com (D.M.); 3 Department of Theriogenology, Faculty of Veterinary Medicine, Cairo University, Giza 12211, Egypt; haneyvet360@yahoo.com; 4 Laboratory of Veterinary Physiology, Tokyo University of Agriculture and Technology, Tokyo 183-8509, Japan; gen@cc.tuat.ac.jp; 5 Laboratory of Veterinary Microbiology, Animal Health Research Institute, Ismailia lab, First District, Ismailia 41522, Egypt; hendabdelmageed312@gmail.com; 6 Laboratory of Veterinary Microbiology, Cooperative Department of Veterinary Medicine, Faculty of Agriculture, Tokyo University of Agriculture and Technology, Tokyo 183-8509, Japan; 7 Department of Pharmacology, Faculty of Veterinary Medicine, Benha University, Moshtohor, Toukh, Elqaliobiya 13736, Egypt; mohamed.elbadawy@fvtm.bu.edu.eg; 8 Department of Pharmacology & Toxicology, College of Pharmacy, King Saud University, Riyadh 11564, Saudi Arabia; rejaie@ksu.edu.sa; 9 Department of Surgery, Anesthesiology and Radiology, Faculty of Veterinary Medicine, Benha University, Moshtohor, Toukh, Elqaliobiya 13736, Egypt; 10 Department of Animal Medicine, Faculty of Veterinary Medicine, Damanhur University, Damanhur, El-Beheira 22511, Egypt; ahmed.elfadadny@vetmed.dmu.edu.eg; 11 Department of Pediatrics and Adolescent Medicine, Juntendo University Graduate School of Medicine, Tokyo 113-8421, Japan; kentaka@juntendo.ac.jp

**Keywords:** Doppler echocardiography, goats, heart, tissue Doppler imaging, xylazine

## Abstract

**Simple Summary:**

Echocardiography, as a feasible tool used to evaluate the cardiac functions, is relatively expanded in the literature of goat’s research because of being a reproducible animal exemplary for cardiovascular research. However, previous studies do not fully describe the full echocardiographic protocol including tissue Doppler imaging (TDI) in goats, the same as used in pet animals, and it was rather mainly focusing on females than males despite its importance for studying the flow dynamics in ruminants. The clinical utility of echocardiography in farm animal practice is still limited and mainly utilized for research purposes. Consequently, the culling strategy of diseased animals suffering from cardiac disorders is usually prioritized more than treatment because of lacking the precise diagnosis of the problem. Breed-specific variations in the echocardiographic measurements may occur and there are no previous study documents the echocardiographic interval parameters in Shiba goats, a native breed in Japan. In the current study, we investigated the feasibility of the full conventional echocardiography protocol as used in companion animal practice and reported for the first time the TDI indices in male goats to provide valuable reference data that can be used for either research or clinical cardiology application in goats. We further highlighted the echocardiographic changes after sedation with xylazine, a common anesthetic medication used in ruminants, as a guide to veterinarians under field conditions.

**Abstract:**

The present study aimed to provide a complete conventional echocardiographic protocol in adult male Shiba goats by using two-dimensional, M-mode, Pulsed Wave Doppler, and tissue Doppler imaging (TDI) echocardiography, and to study concomitantly xylazine-induced alteration of cardiac functions in a highly sensitive species. For this purpose, 12 male Shiba goats were included and complete conventional echocardiography from the standard right and left parasternal views was carried to report the echocardiographic data in male Shiba goats, and also before and after xylazine (Pre-Xyl and Post-Xyl) administration (0.05 mg/IM/kg). Results revealed that the full echocardiographic protocol was feasible in all goats through different cardiac windows and good Doppler alignment was achieved with non-significant variability for assessment of the left ventricular dimensions, trans-pulmonary, trans-aortic, and trans-mitral blood flow. The TDI, which was not reported previously in goats, was successfully assessed from the standard left apical view and showed distinct systolic and diastolic patterns. Xylazine administration was found to significantly reduce heart rate, fractional shortening, and cardiac output as well as the Doppler hemodynamic parameters of the pulmonary artery, aortic and mitral inflows (*p* < 0.05). For TDI, the Post-Xyl group revealed a significant decrease in the myocardial velocities of the septal and lateral wall of the left ventricle. The present study provides, for the first time, complete data of conventional echocardiography in male goats using the full protocol, which is routinely used in pet’s practice. Further, we illustrate in-depth the adverse effect of short-term sedative, xylazine, as used under field conditions and emphasize a simultaneous reduction in both systolic and diastolic cardiac function in goats based on full echocardiography assessment of the heart.

## 1. Introduction

Long-lasting ago, echocardiography became the gold standard and a routine tool used for the diagnostic evaluation of cardiovascular upsets in humans and animals. It also provides non-invasive and reliable landmarks necessary to assess the efficacy, safety, and viability of drugs and the potential risks of their use [1]. Nowadays, various animal models are introduced in cardiovascular diseases (CVD) research including goats. The expansion of using these models aimed at early prediction and effective treatment, which become the major objectives of the cardiologists to limit the epidemic expansion of the CVD in humans and animals [2]. This requires prior validation and establishing the reference intervals of echocardiographic measurements in certain animal species. It is worthy to state that the cardiologist considers goats as an attractive model for experimental human CVD research particularly in certain studies that require fully conscious or exercising animals, flow dynamic research, and atrial fibrillation [3,4,5,6]. Besides, goats are advantageous to other animals because of the easy approach and management, and a relatively reasonable heart and body size comparable to those in humans [7].

Goat’s echocardiography was reported for the first time by Gardner et al. [8] after the diagnosis of tricuspid valve anomaly in male goats. Later on, various studies were proceeding to establish the reference intervals of the cardiac measurements in certain breeds. Notably, the echocardiography in female goats takes a greater fortune of attention than males. In this regard, three quantitative echocardiographic studies have been described in female Saanen goats [9,10,11], and other seldom studies have been undertaken in Swedish goats [12], polish dairy goats [13], and Markhoz Goats [14]. The abovementioned studies investigated the two dimensional (2D) and M-mode echocardiography of the left ventricular functions in healthy adult goats, during pregnancy, lactation, and dry period. Steininger et al. assessed the left ventricle (LV) function in goats before and after isoflurane-induced general anesthesia in goats [11]. Moreover, other comprehensive studies demonstrating quantitative Doppler echocardiography of the pulmonary artery, aorta, and mitral inflow were conducted [9,14,15]. These studies proved that echocardiography is a productive and reliable diagnostic tool in goats like other species. However, previous studies did not provide fundamental data in male goats, despite being an animal of considerable importance in CVD research attraction. Generally, male goats are less included in cardiology research than females; however, the lack of physiological influences like estrous, dry period, pregnancy, and lactation makes the male more advantageous for this purpose. These factors certainly result in fluctuation in the reproductive hormones, cardiac function, and hemodynamic status, which have been documented in women [16,17,18] as well as goats [12,15]. Generally, certain age, breed and sex-specific differences in echocardiographic measurements have been reported in animals. For instance, female whippets had a larger left ventricular diameter in diastole and systole than males [19]. Meanwhile, the breed-specific difference in equine echocardiography was more important than sex [20].

Unlike female goats, quantitative echocardiography in males did not fully described and only two of the aforementioned literature have been conducted in a large group of sheep and goats including male goats without paying any attention to male reference intervals [9,21]. Besides, to our knowledge, there are no comprehensive studies fully investigating the conventional Doppler echocardiography in small ruminants the same as used in pet animal practice. In canine and feline clinics, different echocardiographic techniques are often combined as a complete protocol including mitral inflow and tissue Doppler imaging (TDI) at the mitral annuli, which are important diagnostic indices to evaluate the diastolic function of the left ventricle [22]. On the contrary, the precise involvement of echocardiography in ruminant practice is underutilized and still plays a minor role since cardiac disorders are infrequently detected and the culling strategy of diseased animals is usually prioritized more than medication. Therefore, more echocardiography attentiveness to ruminants, especially the goat is necessary not only for the CVD research model, but also for studying the physiological, pathological, and pharmacological aspects of ruminant species [23].

Under field conditions, xylazine is the most widely used tranquilizer in ruminants for invasive surgery and controlling of seizure animals during examination and transportation. The drug has brought abrupt changes in the cardiopulmonary function including a reduction in the heart rate, respiratory rate, and cardiac output and inducing fluctuation of blood pressure and oxygen tension. These changes have been selectively studied through intra-arterial catheterization, blood gas analysis, M-mode echocardiography, and electrocardiography in different animal species including cattle [24], donkey [25,26], calves [27,28], and goats [29,30]. As far as we concern, exploration of animal models may require anesthesia, but under farm conditions, the effect of short-term tranquilization with xylazine on quantitative echocardiography measurement was not previously studied in goats. Notably, most of the echocardiographic studies in goats serve to measure the conventional systolic LV function; meanwhile, diastolic LV function and regional myocardial function are rarely investigated. Besides, the fortune of quantitative echocardiography in male goats is poorly documented. Therefore, the goal of the current study was to provide complete conventional echocardiographic protocol and study the effect of xylazine administration on cardiac function by using 2D, M-mode, Pulsed–Wave Doppler, and TDI echocardiography as an attempt to provide the normal range of cardiac measurements and to elucidate xylazine-induced alteration of cardiac functions in adult male goats.

## 2. Materials and Methods

### 2.1. Ethical Statement

All experimental procedures and animal care were followed the guidelines established by the local committee belonging to the Tokyo University of Agriculture and Technology, Japan (Tokyo Nokodai, Fuchu, Tokyo, Japan) for the use of animals in experimental studies (Ethical No: 30–78).

### 2.2. Animals

Twelve adult male Shiba goats, 24–28 months old, and 30 ± 3 kg body weight (BW) were enrolled in this study. Goats were housed in a special barn that belongs to the Laboratory of Veterinary Physiology, Tokyo University of Agriculture, Japan, where they received 0.6 kg of hay cubes as a basic diet twice daily and water and trace minerals were kept ad libitum. Detailed physical examination was performed and animals were considered physically fit based on the medical record, auscultation of the heart, electrocardiography and echocardiography, and routine hemato-biochemical profile. In all goats, findings of clinical examination and hemato-biochemical parameters determined before the experiment fell within relevant reference intervals characteristic for the species [31].

### 2.3. Experimental Design

The experiment was conducted to record the normal reference echocardiographic data in male goats as well as before and after sedation with xylazine (Pre-Xyl, Post-Xyl) using a standard dose (0.05 mg/Kg BW/IM, xylazine hydrochloride, Fujita-Pharm, Japan) without any surgical interference according to Grant and Upton [32]. Firstly, a standard xylazine stock solution was prepared using distilled water and the required dose was injected into each animal. The echocardiography was started 15 min after injection when symptoms of tranquilization became prominent.

### 2.4. Conventional Echocardiography

The echocardiography was conducted for wake goats twice at a one-day interval by the same observer to record the full conventional echocardiographic reference data. In the next week, the procedures were repeated twice at a one-day interval to record the echocardiographic data before and after the administration of xylazine. The average of the recorded measurements was obtained from five repeated cardiac cycles. The echocardiographic examination was carried out while animals were restraint by two assistants on a special echocardiographic examination worktable in the lateral recumbency and the forelegs were holding anteriorly as much tolerated by the animal. The right and left precordial areas were shaved and coated with an ultrasonic coupling gel. A ProSound F75 premier CV ultrasonography system (Hitachi Aloka Medical, Tokyo, Japan) supplied with a sector probe of 5 MHz and an electrocardiographic monitoring system was used. The time required from each echocardiographic view was reported. The recorded measurements and image orientation were following the American Society of Echocardiography guidelines [33].

A full conventional two-dimensional, M-mode, and Doppler echocardiography from the standard right and left parasternal views was performed on each animal according to the methods described by De Madron, et al. [34]. Firstly, the right parasternal long axis (RPLAV) four-chamber and five-chamber views were visualized, then the M-mode of the short-axis view at the papillary muscle level was switched on to record the measurements of the LV functions. This includes LV end-diastolic and end-systolic diameters (LVIDd, LVIDs), interventricular septal thickness in diastole and systole (IVSd, IVSs), LV free wall thickness in diastole and systole (LVPWd, LVPWs), cardiac output (CO), stroke volume (SV), fraction shortening (FS), and ejection fraction (EF). After that, the two-dimensional short-axis view at the level of the aorta and main pulmonary artery was obtained and the aortic root diastolic diameter (AoDd), left atrium systolic diameter (LADs), and the left atrium/ aortic diameter ratio (LA/Ao) were reported. Also, the Pulsed-wave Doppler echocardiography (PWDE) was turned on for assessment of the pulmonary artery blood flow with a 2.0 mm sample volume. Later on, the position of animals was changed to obtain the left side echocardiography. From the left apical four-chamber view, the LV diastolic parameters including early (E*_v_*) and late (A*_v_*) velocities, E*_v_*/A*_v_* ratio, and deceleration time (DecT) were obtained from the trans-mitral flow profile using PWDE. Also, the transducer was slightly moved anteriorly to obtain the left apical 5-chamber view for Doppler evaluation of the trans-aortic blood flow.

The following Doppler parameters of the pulmonary and aortic flow has been addressed: peak velocity (PV), pressure gradient (PG), mean velocity (MnV), mean pressure gradient (MPG), velocity-time integral (VTI), right ventricular outflow tract (RVOT), left ventricular outflow tract (LVOT), the cross-sectional area of the artery (CSA), pre-ejection time (PEP), ejection time (ET), and acceleration time (ACCT).

### 2.5. Tissue Doppler Imaging (TDI)

From the left apical 4-chamber view, the tissue Doppler imaging (TDI) of the lateral and septal left ventricular wall was reported by using pulsed-wave TDI echocardiography according to De Madron, et al. [31]. The sample volume was adjusted at 3.0 mm for all goats. The TDI velocity profile includes measurement of the systolic (S_m_) and diastolic indices (E_m_, A_m_, and E_m_/A_m_ ratio) at the point of attachment of the mitral valve to the septal and lateral walls of the LV. The average TDI parameters as well as the ratio between the early diastolic mitral inflow (E*_v_*) and diastolic velocity (E_m_) of the septal and lateral walls of the LV (E*_v_*/E_m_) were calculated. 

### 2.6. Statistical Analysis

The normality test for variable distribution was assessed using the Kolmogorov-Smirnov (KS) test and the coefficient of variations (CV%) between days was calculated. The Paired t-test was used to analyze the significance before and after xylazine administration, with a probability of *p* < 0.05, and data are expressed as Mean ± SEM. For the non-gaussian data which showed a significant KS test, the Mann Whitney test for non-parametric data was used and the median was considered. All statistical analysis was carried out using GraphPad Prism 6.0 (GraphPad Software, San Diego, CA, USA). 

## 3. Results

### 3.1. Clinical Signs

All animals showed prominent signs of sedation 10–15 min after I/M injection including head dropping, ataxia, and limb crossing, hypotonia and hyporeflexia, and mild ruminal bloat. A significant reduction in the respiratory rate (35 ± 6, 18 ± 3; *p* = 0.02) and HR (110 ± 10, 88 ± 7; *p* = 0.03) were observed after 12 min of xylazine administration.

### 3.2. Normality and Variability of the Recorded Echocardiographic Parameters

In the current study, complete conventional echocardiography was done in the standard lateral recumbency from both sides. The technique was verified and different cardiac windows were successfully visualized. The reference interval has been established for all 12 goats and addressing 59 echocardiographic parameters and showing the CV% (Table 1). These data have been obtained from LV function by M-mode, 2D assessment at the aortic and main pulmonary level and PWDE of the pulmonary artery, aorta, and mitral blood flows as well as the pulsed TDI of both septal and lateral wall of the LV.

During the echocardiography examination, no obvious abnormality has been detected in all goats; therefore, all obtained animals have been included in the statistical analysis. The normality test revealed a normal distribution of the majority of the data (*p* > 0.10). However, the PV, MPG, PEP/ET, and ACCT/ET of the aortic and pulmonary Doppler flow, as well as the S_m_ of septal TDI and A_m_ of lateral TDI showed non-Gaussian (*p* < 0.05). The age and BW of goats were normally distributed (*p* > 0.05) with a mean of 26 months and 30 kg, respectively. The CV% for the obtained echocardiographic parameters ranged between 6.6–43%. The data were further classified according to CV% into low (6.6–15%, 20 parameters), moderate (16–25%, 20 parameters), and high (25–40%, 19 parameters) variability. The intra-observer variability of the echocardiographic data, which has been assessed in all goats before xylazine administration showed a non-significance difference (*p* > 0.05). Interestingly, the *p*-value of all tested parameters was exceeding *p* = 0.10. However, certain measurable parameters including AoDd, HR, aortic PV, PG, PEP were trend (*p* = 0.06–0.09).

The required time for a full assessment of different cardiac windows in male goats was estimated as 25–31 min and 17–23 min, respectively in Pre-Xyl and Post-Xyl group excluding the time required for offline measurements.

### 3.3. Right Side Echocardiography and the Effect of Xylazine Administration

**Figure 1:**
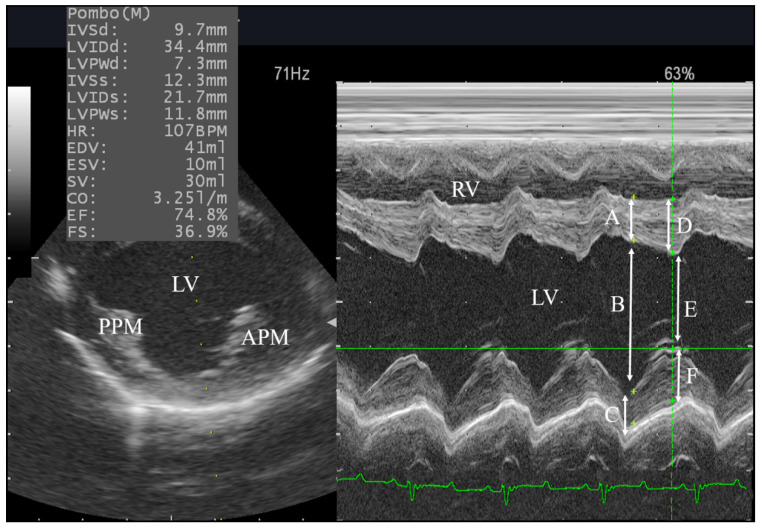
Evaluation of the left ventricle (LV) dimensions in male goats from right parasternal short-axis view (RPSAV) at the papillary muscles level using M-mode echocardiography in lateral recumbency. PPM, posterior papillary muscle; APM, anterior papillary muscle; RV, right ventricle; A, interventricular septum in diastole (IVSd); B, left ventricular internal diastolic diameter (LVIDd); C, left ventricular free wall diastolic diameter (LVFWd); D, interventricular septum in systole (IVSs); E, left ventricular internal systolic diameter (LVISd); F, left ventricular free wall systolic diameter (LVFWs).

**Figure 2:**
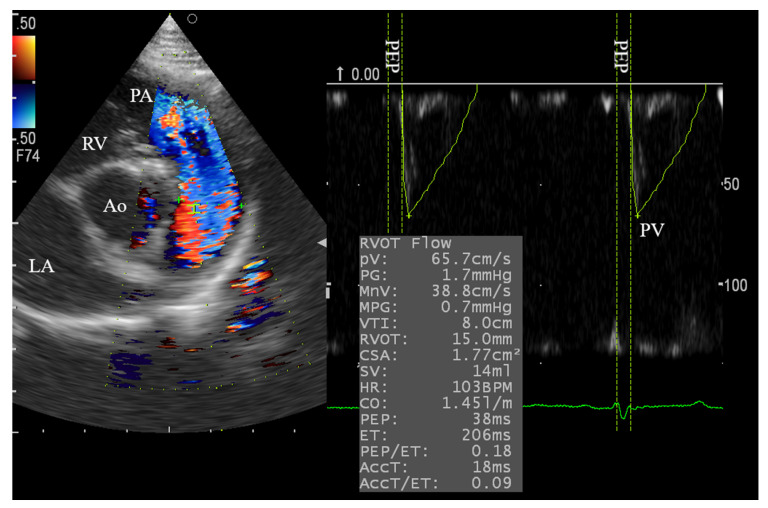
Pulsed-wave Doppler echocardiography for evaluation of the pulmonary blood flows in male goats through RPSAV at the level of the aortic valve and main pulmonary artery. The sample volume was adjusted at 2.0 mm. LA, left atrium; Ao, aorta; RV, right ventricle; PA, main pulmonary artery; PV, peak velocity; PEP, pre-ejection time.

From the right side, the long axis 2D-four and five-chamber views were recognized, which indicated no abnormality and normal Doppler flow in all cardiac chambers. The sector was then oriented to view different short-axis views at the level of the papillary muscle (Figure 1), mitral valve, and aortic and main pulmonary levels (Figure 2). Furthermore, all animals showed good visualization and Doppler alignments to accurately measure the RVOT through PWDE of the pulmonary artery (Figure 2). Approximately, 7–10 min was required to assess the right-side echocardiography, from which the Doppler assessment of the pulmonary artery takes two-thirds of the estimated time to obtain a proper Doppler alignment. The result showed a significant reduction in IVSs, HR, AoDd, LA/Ao ratio as well as the PV, PG, MnV, and MPG of the pulmonary artery (*p* < 0.05) in the Post-Xyl group comparing with the Pre-Xyl group. Left ventricular FS% showed trend reduction (*p* = 0.06) meanwhile the EF% did not change between groups (Table 2).

### 3.4. Left Side Echocardiography

#### 3.4.1. Assessment of the Aortic and Mitral Blood Flow

**Figure 3:**
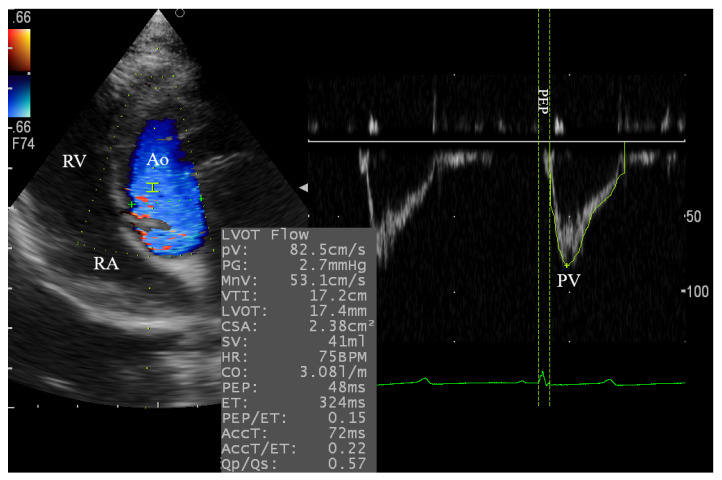
Pulsed wave Doppler echocardiography for evaluation of the aortic blood flow in goats through the left apical 5-chamber view. The sample volume was adjusted at 2.0 mm. Ao, aorta; RA, right atrium; RV, right ventricle; PV, peak velocity; PEP, pre-ejection time.

**Figure 4:**
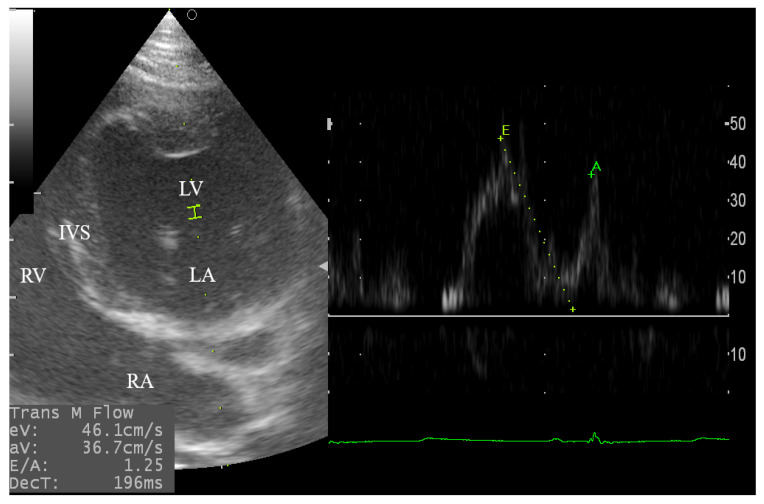
Pulsed wave Doppler echocardiography for evaluation of the mitral inflow in male goats through the left apical 4-chamber view. The sample volume was adjusted at 2.0 mm. The mitral inflow spectral velocity appears as early diastolic wave (E), and late diastolic wave (A) that resemble the atrial contraction. LV, left ventricle; DecT, deceleration time; LA, left atrium; RA, right atrium; RV, right ventricle; IVS, interventricular septum.

The left parasternal apical 2D-four and five-chamber views were relatively difficult to obtain in some cases and required 6–10 min for standard observation and PWDE measurements. The angle was enhanced in some goats by using a pad to lift the animal to a slight extent from its back to obtain the standard view for the successful assessment of the trans-aortic and trans-mitral flows (Figure 3 and Figure 4).

The spectral pattern of the mitral inflow showed variation before and after xylazine administration. In the Pre-Xyl group, the E and A waves were fused in 50% of goats (6 goats), and the E*_v_* was higher than A*_v_* in 4 goats only. On the contrary, the spectral of the E and A were separated in 9 goats in which the E*_v_* was greater than A*_v_* in the Post-Xyl group. Moreover, in the Post-Xyl group, the mitral inflow parameters (E*_v_*, A*_v_*, and DecT) and aortic Doppler parameters (PV, PG, MnV, VTI, LVOT, CSA, CO, ET, PEP/ET, and ACCT) were significantly reduced compared with the Pre-Xyl group (*p* < 0.05) as shown in Table 2.

#### 3.4.2. Tissue Doppler Imaging (TDI) of the Left Ventricular Wall

**Figure 5:**
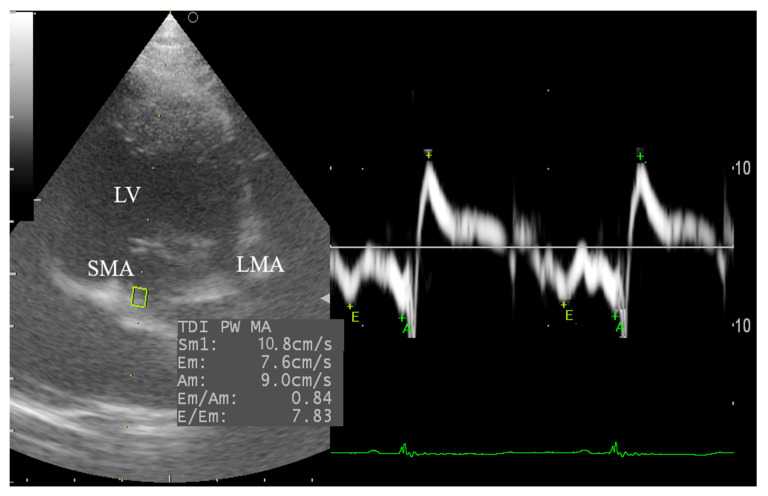
Tissue Doppler imaging (TDI) of male goat’s heart obtained from the left apical 4-chamber view at the septal and lateral mitral annulus (SMA, LMA). The sample volume was adjusted at 3.5 mm. The spectral velocity is expressed as systolic velocity (+, Sm) and two diastolic waves (E, Em; A, Am) which respectively evaluate the early and late diastolic mitral annular velocities.

The pulsed TDI echocardiography was performed in goats after obtaining the left apical four-chamber view. In the Pre-Xyl group, it was difficult to obtain the TDI of the lateral wall of the LV in three goats, which was alternatively obtained from the standard right apical view. The required time to assess the TDI of the lateral and septal left ventricular wall was approximately 6–9 min, and the septal TDI was much easier than the lateral one. Besides, the septal and lateral TDI waveform was varied among groups. The spectral pattern of the TDI was expressed as a positive systolic wave (S_m_) and two retrograde diastolic waves (E_m_ and A_m_) (Figure 5). Although xylazine administration reduces the septal and lateral TDI values, only two goats revealed greater A_m_ than E_m_ before xylazine administration, but after administration 8 goats showed greater A_m_ than E_m_. However, the values of the E_m_, in general, were higher than A_m_ in both groups.

Table 2 illustrates the Pulsed- TDI results in male Shiba goats. The data revealed that the S_m_, E_m,_ and A_m_ of the lateral wall was greater than the septal one (13.5 ± 1.0, 12.5 ± 0.7, 8.5 ± 0.6; 10.6 ± 3.6, 11.5 ± 1.4, 9.8 ± 1.1), respectively. In the Post-Xyl group, there was a significant reduction in the S_m_, A_m_ and E_m_/A_m_ (*p* = 0.003, *p* = 0.001, *p* = 0.048) and trend E*_v_*/E_m_ (*p* = 0.06) of the septal TDI; reduced S_m_ and E_m_ (*p* = 0.00, *p* = 0.03) and trend A_m_ (*p* = 0.06) of the lateral TDI; reduced S_m_ and A_m_ (*p* = 0.00, *p* = 0.02, respectively), and trend E_m_ (*p* = 0.06) of the averaged TDI compared with the Pre-Xyl group.

## 4. Discussion

In cardiology research, the precise details of the heart functions in goats are crucial for the incorporation of the technique into the veterinary practice as well as for effective modeling of cardiology research. In the present study, a full echocardiographic protocol was performed to provide reliable data of the systolic, diastolic, and regional myocardial functions in male Shiba goats, and to explore the detailed impact of xylazine administration on cardiac measurements. Unlike dogs and cats, literature successfully conducts the echocardiography in goats is limited. The presence of an accessory lung right lobe that partially obscuring the heart, gas-filled rumen against the diaphragm, the vertical orientation of the heart, and a large and thick sternum are the main obstacles to obtain good quality images during echocardiography in sheep and goats [35,36,37]. Our research group is qualified in the repetitive echocardiography of small animals as well as goats [23,38]. In the current study, a slight elevation of the animal from its back on the echocardiographic stand with proper orientation of the transducer was required sometimes for better alignment to measure the mitral inflow, aortic flow as well as the TDI from the left apical view. However, assessment of the left apical views and TDI are time-consuming, especially before xylazine administration because of the movement of animals and different cardiac orientation [10].

Importantly, few studies are focusing on goat’s echocardiography in which female goats, particularly the Saanen breed, are the most studied animal [10,13]. Meanwhile, Shiba goats; a native Japanese non-seasonal breeding animal [39,40] was not investigated, despite being an efficient experimental model for characterization of the blood flow dynamics in ruminants [41,42,43]. To our knowledge, only one reputable study has been conducted on a group of Golden Guernsey and Saanen goats from both sex but the authors did not specify any results for male goats which is inevitable to get accurate echocardiographic reference intervals [21]. Doppler echocardiography is routinely used to diagnose flow dynamics in most species. In this study, the recorded pulmonary, aortic, and mitral inflow assessed by PWDE were of high quality and indicates a good alignment of the transducer with the blood flow [14]. Doppler evaluation of the blood flow across these valves is fairly reported in goats and our results showed that a standard and good quality PWDE could be obtained on recumbent fully conscious, and sedated goats without documenting any flow abnormalities [34]. Also, the reported Doppler parameters were consistent with previously reported pulmonary and aortic flow measurements in goats [9,15]. Since the cardiac orientation and successful rate to assess different cardiac windows in previous studies were relatively different, therefore, attention should be paid to consider the method used for the measurement of cardiac parameters. Besides, the applicability of echocardiography in ruminant species still limited. Thus, the aforementioned studies mainly describe the echocardiography reference data in goats under normal physiologic states and anesthesia.

In the present study, the reported echocardiographic indices are in good agreement with previously reported indices of LV M-mode echocardiography of Saanen goats with an average BW of 66 ± 9 kg [9], Golden Guernsey and Saanen goats at average BW of 48 ± 10 Kg [13,21] and growing sheep [35]. In the current study, the BW of male goats was normally distributed and we did not examine the effect of BW on the obtained echocardiographic parameters. Nevertheless, a significant correlation between BW and LV M-mode echocardiography in Pantja goats was observed [44].

TDI is widely used in canine and feline as well as human cardiology to assess the global and regional wall motion velocity during systole and diastole at the two points of the mitral annulus and independently from loading conditions [34,45]. To the best of our knowledge, this is the first study to describe the TDI in goats as obtained by pulsed TDI. In the present study, the septal and posterior wall spectral velocities are expressed as positive systolic wave (S_m_, movement of the mitral annulus toward the cardiac apex) and two negative diastolic velocities; E_m_ and A_m_, which are related to myocardial relaxation and atrial contraction, respectively. Unfortunately, there are no data in the previously cited studies in ruminant species to compare the TDI with our results. In sheep, the TDI was previously obtained from RPSAV at the mitral valve level [46]. In the current study, the LV septal and lateral wall motion were expressing the same patterns as humans [45], dogs and cats [34], and rabbits [47,48]. The changes in the TDI spectral pattern before and after xylazine administration were related to changes in the E and A spectral of the mitral inflow. In this concern, E and A waves of the mitral inflow were fused and E*_v_* was higher than A*_v_*, and the E_m_ was higher than A_m_ in both groups. Meanwhile, reduced myocardial contractility and HR were observed in the Post-Xyl group with unfused mitral inflow E and A waves. This could be attributed to the direct negative chronotropic effect of xylazine, which results in the reduction of myocardial contractility, mitral inflow, and TDI parameters [48,49,50] and therefore results in a significant reduction in the diastolic function.

The mitral inflow and TDI velocities are useful indices for the interpretation of the diastolic function [51]. Generally, the E_m_ velocity is negatively correlated with early diastolic pressure thereby considered as an indicator for relaxation and elastic recoil of the LV. Likewise, the elevation of the LV filling pressure results in increased mitral E*_v_* and concomitant reduction in E_m_. Meanwhile, the E*_v_*/E_m_ ratio is of particular value to interpret the congestive states since it correlates well with the LV end-diastolic pressure or pulmonary capillary wedge pressure [45]. Practically, the ratio between the E*_v_*/E_m_ and E_m_/A_m_ is indicative of congestion when exceeding 15 and 1.5, respectively in dogs and cats [34] but there is no gold standard for mitral inflow and TDI indices in small ruminants.

To the best of our knowledge, there has been in international literature no reporting quantitative echocardiographic data in awake versus tranquilized goats using our approach. Xylazine, as a gold standard α2 agonist, is the most trustworthy drug used in farm animal practice for sedative and analgesic purposes [50]. In the current study, short-term sedation has been conducted to mimic the field condition of minimal interference. Our results did not document any structural or flow abnormalities among groups. Nevertheless, various physiological parameters in M-mode, PWDE, and pulsed TDI were changed after xylazine administration. In this regard, xylazine administration results in a reduction in the IVSs, HR, AoDd, LA/Ao, and Doppler parameters of the pulmonary, aorta, and mitral valves. A combination of xylazine with ketamine reduces FS, EF, and CO and increases the LV systolic and diastolic dimensions in the laboratory animal models [48,52], and sheep [35]. Also, In the present study both E*_v_* and A*_v_* were significantly reduced in the Post-Xyl group (55.2 ± 3.6, 52.4 ± 4.1) compared with the Pre-Xyl group (45.8 ± 2.4, 39.1 ± 2.4) with approximately the same value which results in a non-significant change in the E/A ratio. Also, xylazine administration significantly reduced the atrial contractility as observed by a significant reduction in A*_v_* and A_m_. The net effect of xylazine on systolic and diastolic functions of the LV might depend on its ability to induce changes in preload, afterload, contractility, and heart rate. Therefore, the reduction in systolic FS and maintenance of CO after xylazine administration might be attributed to alterations in loading conditions and the chronotropic activity [48,53].

In the present work, some potential limitations deserve attention. We did not compare our results with those reported in female goats because of the difference in the used procedures and protocol. For example, some studies monitor only the LV function by M-mode [10], others conducting Doppler echocardiography in both sexes but did not provide selective data for male goats [9]. Another limitation of the present study is the use of xylazine for sedation, but it was selected carefully since it is widely used in ruminant practice with a well-known cardio-depressant effect that is used to investigate diastolic indices and the TDI changes. Also, previous studies reported the dose-response effect of xylazine administration on cardiac function using other protocols for evaluation of heart functions, which differ from our plan, and further study is required to investigate the dose-response effect of xylazine using full echocardiography protocol.

## 5. Conclusions

The present work provides, for the first time, a detailed description of echocardiographic measurements in healthy male Shiba goats by using various echocardiographic techniques including 2-D, M-mode, Doppler Echocardiography, and pulsed TDI. This is considered a promising step forward of the used echocardiographic approaches for diagnostic and prognostic evaluation of cardiac functions in goats for further clinical and research purposes. Besides, we highlighted the effect of xylazine administration on cardiac parameters in goats that revealed a rapid reduction in the cardiac parameters even under the recommended dosing.

## Figures and Tables

**Table 1:** Reference interval, normality, and variability of the full conventional echocardiographic measurements in male Shiba goats.

Assessment	Parameters	Unit	Mean	SD	Normality		CV%
					*P*-vlue	Summary	
LV measurements	IVSd	mm	8.084	1.218	>0.10	ns	15.00
	LVIDd	mm	33.1	2.896	>0.10	ns	8.75
	LVPWd	mm	7.944	1.369	>0.10	ns	17.24
	IVSs	mm	12.32	0.819	>0.10	ns	6.65
	LVISd	mm	21.09	2.454	>0.10	ns	11.64
	LVPWs	mm	12.36	1.948	0.052	ns	14.76
	HR	pbm	106.5	10.67	>0.10	ns	10.02
	EDV	mL	37.15	9.268	>0.10	ns	24.95
	ESV	mL	ml	3.106	>0.10	ns	30.91
	EF	%	70.28	7.017	>0.10	ns	9.99
	FS%	%	35.45	4.157	>0.10	ns	11.73
LA/Ao ratio	LADs	mm	29.4	2.801	>0.10	ns	9.53
	AoDd	mm	19.36	2.078	>0.10	ns	10.73
	LA/Ao		1.513	0.234	>0.10	ns	14.46
Pulmonary	PV	cm/s	81.34	14.64	0.063	ns	17.99
	PG	mmHg	2.561	0.574	>0.10	ns	22.43
	MnV	cm/s	55.43	11.37	>0.10	ns	20.51
	MPG ^#^	mmHg	1.427	0.652	0.0021	**	40.01
	VTI	cm	11.77	2.571	>0.10	ns	21.85
	RVOT	mm	14.63	2.181	>0.10	ns	14.91
	CSA	cm^2^	1.721	0.495	>0.10	ns	28.78
	PEP	ms	42.04	5.684	>0.10	ns	13.52
	ET	ms	212.8	29.56	>0.10	ns	13.89
	PEP/ET ^#^		0.201	0.051	0.0001	***	24.38
	ACCT	ms	93.77	33.5	>0.10	ns	35.73
	ACCT/ET ^#^		0.427	0.138	0.016	*	32.19
Aortic	PV	cm/s	81.75	10.62	0.064	ns	13.00
	PG ^#^	mmHg	2.781	0.757	0.006	**	27.21
	MnV	cm/s	51.75	7.118	>0.10	ns	13.75
	VTI	cm	12.42	2.558	>0.10	ns	20.59
	LVOT	mm	16.22	2.552	>0.10	ns	13.73
	CSA	cm^2^	2.126	0.635	>0.10	ns	29.86
	SV	mL	27.14	11.73	>0.10	ns	39.81
	HR	bpm	106.8	18.19	>0.10	ns	17.02
	CO	L/m	2.873	1.237	>0.10	ns	39.05
	PEP	ms	38.86	10.98	>0.10	ns	28.25
	ET	ms	240.2	41.22	>0.10	ns	17.16
	PEP/ET ^#^		0.167	0.072	0.005	**	39.95
	ACCT	ms	67.48	18.68	>0.10	ns	27.69
	ACCT/ET ^#^		0.282	0.082	0.044	*	29.09
Mitral inflow	E*_v_*	cm/s	48.71	8.872	>0.10	ns	18.21
	A*_v_*	cm/s	52.08	9.816	>0.10	ns	18.85
	E/A		0.944	0.196	>0.10	ns	20.78
	DecT	ms	126.9	26.41	>0.10	ns	20.81
Septal TDI	S_m_^#^	cm/s	10.61	1.006	0.041	*	9.48
	E_m_	cm/s	7.852	1.642	>0.10	ns	20.91
	A_m_	cm/s	8.447	2.465	>0.10	ns	29.19
	E_m_/A_m_		1.024	0.375	>0.10	ns	36.60
	E*_v_*/E_m_		6.548	1.087	>0.10	ns	16.60
Lateral TDI	S_m_	cm/s	13.52	2.974	>0.10	ns	22.01
	E_m_	cm/s	10.76	3.019	>0.10	ns	28.06
	A_m_^#^	cm/s	9.978	2.167	0.002	**	21.72
	E_m_/A_m_		1.122	0.372	>0.10	ns	33.14
	E*_v_*/E_m_		4.938	1.251	>0.10	ns	23.34
Average TDI	S_m_	cm/s	12.06	1.505	>0.10	ns	12.47
	E_m_	cm/s	11.15	1.228	>0.10	ns	11.01
	A_m_	cm/s	10.66	3.06	>0.10	ns	28.70
	E_m_/A_m_		1.114	0.442	>0.10	ns	39.66
	E*_v_*/E_m_		5.687	1.434	>0.10	ns	24.92

Echocardiographic parameters were obtained from the full assessment protocol (2D, M-mode, PW Doppler, TDI) in male goats. Values are expressed as mean ±SD (*n* = 12). *, **, *** fitted to compare the significance (*p* < 0.05, 0.01, 0.001), respectively. ns, non significant. ^#^ used to express the median for non-Gaussian data. CV%, coefficient of variation; IVSd, interventricular septum diastolic diameter; LVIDd, left ventricular (LV) internal diastolic diameter; LVFWd, LV free wall diastolic diameter; IVSs, interventricular septum systolic diameter; LVISd, LV internal systolic diameter; LVFWs, LV free wall systolic diameter; EDV, LV end-diastolic volume; ESV, LV end-systolic volume; EF, ejection fraction; FS, fraction shortening; LADs, left atrium systolic diameter; AoDd, aortic diastolic diameter; LA:Ao, left atrial to aortic diameter ratio; PV, peak velocity; PG, pressure gradient; MnV, mean velocity; MPG, mean pressure gradient; VTI, velocity-time integral; RVOT, right ventricular outflow tract; HR, heart rate; SV, stroke volume; Co, cardiac output; LVOT, LV outflow tract; CSA, cross-sectional area; PEP, pre-ejection time; ET, ejection time; ACCT, acceleration time; E_v_, early diastolic velocity mitralis, A*_v_*, late (atrial) flow velocity mitralis; E/A, early to ate mitral inflow velocity ratio; DecT, deceleration time; S_m_, systolic velocity of the LV wall; E_m_, early diastolic velocity of the LV wall; A_m_; late diastolic velocity of the LV wall; E_m_/A_m_, Early to late diastolic velocity of the LV wall; E*_v_*/E_m_, early diastolic velocity mitralis to early diastolic velocity of the LV wall ratio.

**Table 2:** Two-dimensional, pulsed-wave Doppler, and tissue Doppler imaging echocardiography measurements before and after xylazine administration in male Shiba goats.

Assessment	Unit	Pre-Xyl	Post-Xyl	*P*-value
LV measurements				
IVSd	mm	7.5 ± 0.5	8.0 ± 0.2	0.38
LVIDd	mm	33.6 ± 1.4	34.4 ± 1.1	0.64
LVPWd	mm	7.9 ± 0.6	7.2 ± 0.3	0.24
IVSs	mm	13.3 ± 0.2	12.3 ± 0.2	0.02 *
LVISd	mm	19.3 ± 1.5	21.3 ± 0.9	0.15
LVPWs	mm	13.0 ± 0.7	12.8 ± 0.7	0.73
EDV	mL	39.5 ± 4.8	41.7 ± 3.9	0.72
ESV	mL	8.7 ± 1.7	10.1 ± 1.3	0.30
EF	%	75.7 ± 4.6	74.2 ± 2.9	0.71
FS%	%	41.5 ± 2.9	37.8 ± 2.6	0.07
LA/Ao ratio				
LADs	mm	29.8 ± 0.9	33.1 ± 3.1	0.29
AoDd	mm	20.2 ± 0.5	18.5 ± 0.8	0.02 *
LA/Ao		1.5 ± 0.1	1.7 ± 0.1	0.04 *
Pulmonary Doppler				
PV	cm/s	85.4 ± 4.0	70.7 ± 3.7	0.04 *
PG	mmHg	3.0 ± 0.3	2.1 ± 0.2	0.04 *
MnV	cm/s	56.2 ± 2.8	46.0 ± 3.3	0.07
MPG ^#^	mmHg	1.4 ± 0.1	1.0 ± 0.1	0.04 *
VTI	cm	11.3 ± 0.5	11.0 ± 1.5	0.88
RVOT	mm	14.6 ± 0.5	15.7 ± 0.6	0.17
CSA	cm *	1.7 ± 0.1	2.0 ± 0.1	0.15
PEP	ms	42.6 ± 3.3	45.0 ± 2.7	0.49
ET	ms	205.2 ± 12.6	234.2 ± 22.4	0.32
PEP/ET ^#^		0.2 ± 0.0	±0.2 ± 0.0	0.45
ACCT	cm/s	72.5 ± 11.8	92.3 ± 12.9	0.35
ACCT/ET ^#^		0.3 ± 0.0	0.4 ± 0.0	0.18
Aortic Doppler				
PV	cm/s	93.1 ± 3.9	78.3 ± 1.5	0.01 *
PG ^#^	mmHg	3.4 ± 0.3	2.4 ± 0.1	0.00 *
MnV	cm/s	55.9 ± 3.3	49.0 ± 0.8	0.05
VTI	cm	11.9 ± 0.8	14.5 ± 0.8	0.01 *
LVOT	cm	13.8 ± 0.6	16.6 ± 0.4	0.02 *
CSA	cm^2^	1.5 ± 0.1	2.2 ± 0.1	0.01 *
SV	mL	31.1 ± 2*1	30.7 ± 2.3	0.70
HR	bpm	128.5 ± 7.5	85.9 ± 6.3	0.01 *
CO	L/m	3.8 ± 0.37	2.7 ± 0.2	0.02 *
PEP	ms	35.3 ± 4.8	45.4 ± 3.3	0.11
ET	ms	216.0 ± 16.9	294.8 ± 15.7	0.00 *
PEP/ET ^#^		0.20 ± 0.0	0.20 ± 0.0	0.81
ACCT	cm/s	56.7 ± 6.1	72.4 ± 3.9	0.02 *
ACCT/ET ^#^		0.25 ± 0.0	0.25 ± 0.0	0.81
Mitral inflow				
E*_v_*	cm/s	55.2 ± 3.6	45.8 ± 2.4	0.05 *
A*_v_*	cm/s	52.4 ± 4.1	39.1 ± 2.4	0.01 *
E/A		1.0 ± 0.1	1.2 ± 0.1	0.15
DecT	ms	106.5 ± 13.4	163.4 ± 15.2	0.02 *
Pulsed-TDI				
S_m_ Sept ^#^	cm/s	10.5 ± 0.36	8.25 ± 0.38	0.00 *
E_m_ Sept	cm/s	8.5 ± 0.6	8.4 ± 0.4	0.97
A_m_ Sept	cm/s	9.8 ± 1.1	6.7 ± 0.6	0.03 *
E_m_/A_m_ Sep		1.0 ± 0.2	1.3 ± 0.1	0.04 *
E*_v_*/E_m_ Sept		6.7 ± 0.6	5.6 ± 0.3	0.06
S_m_ Lat	cm/s	13.52 ± 1.05	9.48 ± 0.66	0.00 *
E_m_ Lat	cm/s	12.5 ± 0.4	10.7 ± 0.5	0.03 *
A_m_ Lat ^#^	cm/s	11.4 ± 1.4	8.6 ± 0.6	0.23
E_m_/A_m_ Lat		1.3 ± 0.2	1.3 ± 0.1	0.81
E*_v_*/E_m_ Lat		4.6 ± 0.5	4.5 ± 0.3	0.83
Average TDI				
S_m_	cm/s	12.06 ± 0.53	8.79 ± 0.48	0.00 *
E_m_	cm/s	11.15 ± 0.43	9.57 ± 0.42	0.06
A_m_	cm/s	10.66 ± 1.08	7.66 ± 0.48	0.02 *
E_m_/A_m_		1.11 ± 0.16	1.32 ± 0.12	0.18
E*_v_*/E_m_		5.69 ± 0.51	5.05 ± 0.24	0.28

Assessment of heart functions using a full conventional echocardiographic protocol in male Shiba goats before and after sedation. Values are expressed as mean ± SEM (*n* = 12). ^#^ used to express the median for non-Gaussian data. * fitted to compare the significance (*p* < 0.05), respectively. IVSd, interventricular septum diastolic diameter; LVIDd, left ventricular (LV) internal diastolic diameter; LVFWd, LV free wall diastolic diameter; IVSs, interventricular septum systolic diameter; LVISd, LV internal systolic diameter; LVFWs, LV free wall systolic diameter; EDV, LV end diastolic volume; ESV, LV end-systolic volume; EF, ejection fraction; FS, fraction shortening; LADs, left atrium systolic diameter; AoDd, aortic diastolic diameter; LA/Ao, left atrium to aortic diameter ratio; PV, peak velocity; PG, pressure gradient; MnV, mean velocity; MPG, mean pressure gradient; VTI, velocity time integral; RVOT, right ventricular outflow tract; HR, heart rate; SV, stroke volume; Co, cardiac output; LVOT, LV outflow tract; CSA, cross sectional area; PEP, pre-ejection time; ET, ejection time; ACCT, acceleration time; E_v_, early diastolic velocity mitralis, A*_v_*, late (atrial) flow velocity mitralis; E/A, early to ate mitral inflow velocity ratio; DecT, deceleration time; S_m_, systolic velocity of the LV wall; E_m_, early diastolic velocity of the LV wall; A_m_; late diastolic velocity of the LV wall; Sep, septal wall; Lat, lateral wall; E_m_/A_m_, Early to late diastolic velocity of the LV wall; E*_v_*/E_m_, early diastolic velocity mitralis to early diastolic velocity of the LV wall ratio.

## Abbreviations List

ACCT	Acceleration time
ACCT/ET	Acceleration time/ejection time
A_m_	Late diastolic velocity of the LV wall
AoDd	Aortic diastolic diameter
A*_v_*	Late diastolic velocity mitralis
CO	Cardiac output
CSA	Cross-sectional area
DecT	Deceleration time
E/A	E/A, early to late mitral inflow velocity ratio;
E/E_m_	Early diastolic velocity mitralis/early diastolic velocity of the LV wall
EDV	LV end-diastolic volume
EF	Ejection fraction
E_m_	Early diastolic velocity of the LV wall
E_m_/A_m_	Early to late diastolic velocity of the LV wall
ESV	LV end-systolic volume
ET	Ejection time
E*_v_*	Early diastolic velocity mitralis
FS%	Fraction shortening
HR	Heart rate
IVSd	Interventricular septum diastolic diameter
IVSs	Interventricular septum systolic diameter
LA/Ao	Left atrial/aortic diameter
LADs	Left atrium systolic diameter
LV	Left ventricle
LVIDd	LV internal diastolic diameter
LVISd	LV internal systolic diameter
LVOT	LV outflow tract
LVPWd	LV free wall diastolic diameter
LVPWs	LV free wall systolic diameter
MnV	Mean velocity
MPG	Mean pressure gradient
PEP	Pre-ejection time
PEP/ET	Pre-ejection time/ejection time
PG	Pressure gradient
PV	Peak velocity
PWDE	Pulsed-wave Doppler echocardiography
RVOT	Right ventricular outflow tract
S_m_	Systolic velocity of the LV wall
SV	Stroke volume
TDI	Tissue Doppler imaging
VTI	Velocity-time integral
